# Beyond chemotherapy: Exploring tree turmeric root and nano-hydroxyapatite for neuroprotective applications

**DOI:** 10.22038/ijbms.2025.84185.18205

**Published:** 2025

**Authors:** Chuangen Li, Sriram Kaliamoorthy, Mariappan Vijayalakshmi

**Affiliations:** 1 Department of Rehabilitation Medicine, Ankang People’s Hospital, Ankang, 725000, China; 2 Vinayaka Mission’s Medical College and Hospital, Vinayaka Mission’s Research Foundation (Deemed to be University), Karaikal, Puducherry, India, 609609; 3 Department of Chemistry, Nandha Engineering College, Tamil Nadu- 638052, India

**Keywords:** Antioxidant, Hydroxyapatite Nanoparticles, Neuropathic pain, Tree turmeric root

## Abstract

**Objective(s):**

To investigate the physicochemical properties, in vitro efficacy, and in vivo therapeutic potential of novel tree turmeric root and nano-hydroxyapatite (TRE@NHA) composites in mitigating chemotherapy-induced peripheral neuropathy (CIPN).

**Materials and Methods:**

TRE@NHA composites were synthesized and characterized using FTIR, XRD, TGA, and HRTEM. In vitro studies using PC12 cells assessed cytotoxicity, anti-inflammatory effects, and neuroprotective properties. An in vivo rat model of CIPN was established using paclitaxel (PTX). Behavioral assessments, histopathological analysis, and oxidative stress markers were evaluated in sciatic nerve tissues.

**Results:**

TRE@NHA composites demonstrated successful integration of TRE into the NHA matrix. In vitro studies revealed significant anti-inflammatory and neuroprotective effects of TRE@NHA-2, particularly in suppressing cytokine production, enhancing cell viability, and mitigating oxidative stress. *In vivo*, TRE@NHA-2 effectively alleviated PTX-induced neuropathic pain, reduced neuronal damage, and exhibited potent antioxidant properties.

**Conclusion:**

This study demonstrates the successful development and characterization of novel TRE@NHA composites. The findings strongly suggest that TRE@NHA-2 possesses promising therapeutic potential for mitigating CIPN due to its anti-inflammatory, antioxidant, and neuroprotective properties.

## Introduction

Chemotherapy prolongs the lifespan of patients; however, it also comes with various side effects, such as neuropathic pain. Chemotherapy, particularly from taxane- and platinum-based anticancer drugs, frequently results in nerve damage, referred to as chemotherapy-induced peripheral neuropathy (CIPN), which often results in the discontinuation of treatment (1-3). CIPN manifests as sensory loss, tingling, difficulty walking, discomfort, abnormal sensations, numbness, and increased sensitivity to touch. Treatment options for CIPN are limited and may have undesirable side effects (4-5). Doctors often prescribe tricyclic antidepressants and anticonvulsants for individuals with CIPN, even though their effectiveness is limited and they can cause unwanted side effects.

The American Society of Clinical Oncology (ASCO) supports duloxetine for CIPN; however, studies have indicated that it may cause additional harmful effects, including brain toxicity, which can result in reduced reflexes, confusion, and catatonia (6). Traditional nonsteroidal anti-inflammatory medications and opioids, although they possess antiallodynic properties, are regarded as undesirable due to their adverse effects, including seizures and respiratory dysfunction. Thus, safer alternative treatments for CIPN are necessary.

Traditional medicinal systems have recognized the pain-relieving properties of various plants, which numerous independent studies have confirmed. These plants are *Acorus calamus, Aconiti tuber, Allium sepa, Allium sativum, Artemisia dracunculus, Cannabis sativa, Emblica officinalis, Ginkgo biloba, Nigella sativa, Curcuma longa, *and* Vochysia divergens* (7-9). Among these herbs, Berberis aristata, also called tree turmeric, is a well-known plant species (10). This plant has been highly valued in Ayurvedic medicine for its numerous therapeutic properties for millennia. For a long time, individuals have utilized various parts of plants, such as stems, roots, and bark, to address a diverse array of medical issues. Pharmacological studies have confirmed the traditional use of tree turmeric, demonstrating its notable anti-inflammatory, antidiabetic, anticancer, and antimicrobial effects (11-12). Various plant phytochemicals, such as alkaloids, flavonoids, and tannins, have generated significant interest in contemporary medicine. Recent studies indicate turmeric has strong anti-inflammatory, anti-oxidant, and pain-relieving properties (13-14).

This study aims to examine the characteristics of tree turmeric roots combined with nanoparticles both *in vitro* and *in vivo*. The findings emphasize the promise of this combination as a therapeutic approach for neuropathic pain. Nanoparticles possess unique properties that can assist in addressing challenges related to the delivery of medications to the peripheral nervous system. Nanohydroxyapatite (NHA) is a biocompatible and osteoconductive material with significant potential for application in drug delivery systems across various medical fields (15, 16). NHA, the primary mineral component of bone, possesses unique characteristics that render it an attractive choice for drug delivery. The primary mineral found in bone, NHA, has unique properties that render it an attractive choice for drug delivery. NHA holds considerable promise as a versatile basis for creating innovative drug delivery systems to address neuropathic pain (17).

A meticulously regulated factor might avert chemotherapy-induced peripheral neuropathy. This study explored if TRE@NHA can reduce neuropathic pain in a mouse paclitaxel-induced nerve damage (CIPN) model. The PTX dose consistently induced symptoms of CIPN, including sensory loss and discomfort, as previously described (18). When PTX activates CIPN, it results in oxidative stress that deteriorates mitochondrial function. This, in turn, produces effects that relieve pain. In chemotherapy-induced peripheral neuropathy (CIPN), oxidative stress and inflammation interact and mutually affect one another simultaneously (19). Chemotherapy induces alterations in the immune system that result in inflammation within the nervous system during the progression of CIPN. Thus, herbs that lower oxidative stress and inflammation while modulating the immune system are highly effective in combating CIPN (20-21). Therefore, this research shows that TRE@NHA effectively enhances the pathophysiology of CIPN by addressing various mechanisms involved in the disease process. TRE@NHA is a promising approach to address neuropathic pain issues as it reduces neuroinflammation, alters nociception, and accelerates the degeneration of axons resulting from oxidative stress.

## Materials and Methods

### Preparation of TRE@NHA

We synthesized NHA using a slightly modified version of a previously documented procedure (22). A 1 M solution of calcium chloride (CaCl₂)(Sigma Aldrich) was gradually introduced into a 0.6 M solution of phosphoric acid (H₃PO₄) (Sigma Aldrich) while stirring continuously. The pH of the reaction mixture was adjusted to 10 using a NaOH solution (Sigma Aldrich). The solution was then agitated at ambient temperature for 24 hr to ensure complete precipitation. The resulting precipitate was isolated from the supernatant via centrifugation (Sigma Laboratory Centrifuges-5000 RPM) and filtering (Whatman filter paper-Sigma Aldrich). The collected precipitate was subjected to drying in a muffle furnace (BIOBASE-1000 ^°^C) at 700 ^°^C. After drying, we finely crushed the NHA powder and stored it in a Tarson desiccator for further examination.

We obtained the tree turmeric root extract (TRE) powder from a nearby Ayurvedic store. TRE and hydroxyapatite (NHA) nanoparticles were mixed via a mortar-and-pestle grinding process to create TRE@NHA composites. Two distinct formulations of TRE@NHA were created: TRE@NHA-1, which consisted of a 1:1 ratio of 1 mg of TRE to 1 mg of NHA, and TRE@NHA-2, which had a 2:1 ratio of 2 mg of TRE to 1 mg of NHA. These formulations were later assessed for their potential use in treating neuropathic pain.

### Materials characterization

Various analytical methods were used to characterize the samples. FTIR analysis was performed via a Shimadzu-IRSpirit-X Series instrument to identify the functional groups in the samples. XRD patterns were used to analyze the crystalline structure and phase purity with a D8 Endeavor Bruker diffractometer. The samples’ thermal stability and decomposition behavior were assessed via TGA via a PerkinElmer TGA 8000™ instrument. The shape, size distribution, and structural characteristics of the nanoparticles were examined with HRTEM using an FEI TECNAI G 2-20 TWIN microscope.

### In vitro cytotoxic and anti-inflammatory assays

PC12 cells were used to evaluate the TRE@NHA composites’ cytotoxic and anti-inflammatory qualities. After undergoing differentiation, the cells were stimulated with LPS to start an inflammatory response. The MTT test was used to assess the cell viability, and the enzyme-linked immunosorbent assay (ELISA) was used to measure the secretion levels of interleukin-1β (IL-1β). Furthermore, the effect of the TRE@NHA composite on TNF-α-induced SEAP production was evaluated in TNF-α cells. The cells were subjected to varying concentrations of the TRE@NHA composites for twenty-four hours. The cells were then activated with either TNF-α or LPS. The amounts of SEAP and IL-1β that were released were then measured using QUANTI-Blue™ solution and ELISA, respectively. The cell cultures were maintained at 37 ^°^C with a 5% CO_2_ concentration in a humidified incubator.

### AO/PI dual-staining assay

Following a 24-hour incubation period, the PC12 cells were allowed to proliferate in a 96-well plate at a density of 103 cells per well. The cells were then exposed to the TRE@NHA-1 and TRE@NHA-2 composites for 24 hr at 50 and 100 μg/ml doses, either with or without 500 ng/ml LPS. Following treatment, the cells were incubated for 20 min at 37 ^°^C after exposure to a 1:1 μg/ml concentration of AO/PI. An LFM-C10 fluorescence microscope was used to take fluorescence pictures after three PBS washes.

### DAPI and Rhodamine123 staining

PC12 cells were cultured in a 96-well plate at a density of 10^3 cells per well and incubated overnight. The cells were then treated with TRE@NHA-1 and TRE@NHA-2 composites (at concentrations of 50 or 100 μg/ml) in the presence or absence of LPS (at a concentration of 500 ng/ml) for 24 hr. Following treatment, the cells were subjected to a concentration of 1 μg/ml DAPI or rhodamine 123 and incubated for 20 min in a dark environment at 37 °C. After three washes with PBS, the cells were treated with a 1% paraformaldehyde solution for fixation. Fluorescence images were obtained using an LFM-C10 fluorescence microscope (23). The *in vivo* study, pain behavior, and histopathological evaluation procedures are detailed in the supplementary information.

### In vivo study

The animal studies were conducted following the research protocol ethical number (File No: 2023-89), which obtained approval from the Institutional Animal Ethical Committee (IAEC) of the Department of Rehabilitation Medicine, Ankang People’s Hospital, China. The male mice used in the study were aged between 5 and 8 weeks and weighed between 22 and 25 grams. They were maintained in a controlled environment with a temperature of 23±4 ^°^C, relative humidity ranging from 50% to 70%, and a light-dark cycle of 12 hr of light followed by 12 hr of darkness. The mice had unrestricted access to a standard pellet meal and sterile filtered reverse osmosis water.

### CIPN mice model

Male mice aged 6 to 9 weeks were intentionally administered chemotherapy-induced peripheral neuropathy (CIPN) using PTX (Sigma Aldrich), following a method outlined in previous studies (24, 25). Animals in group one received normal saline (Sigma Aldrich) orally and acted as the control group (NC). Groups two through five received intraperitoneal (IP) injections of 2.5 mg/kg body weight of PTX for six consecutive days. After evaluating neuropathic pain, the test subjects were randomly assigned to therapy groups of three to five. The disease control group two was administered Na-CMC (0.3%)(Sigma Aldrich) orally. The reference group three received intraperitoneal injections of pregabalin (Sigma Aldrich) at 100 mg/kg body weight. Groups four and five received oral doses of TRE@NHA-2 at 50 mg/kg and 100 mg/kg, respectively. Each group contained six animals, and treatments were given daily for 18 days.

### Evaluation of pain behaviors

*Tail flick test*

A test was performed to assess the duration of the reaction time to nociceptive discomfort. The experiment used a PHA16401 Analgesiometer Tail Flick device with an infrared radiation intensity of fifty. The technique employed was a revised approach documented earlier (26). The measurements for each animal were taken three times, with a five-minute interval between each measurement. A threshold of fifteen seconds was used. The study was conducted on days 0, 6, 12, and 18. 

*Hot plate test*

The hot plate test, designed to measure response time, was carried out on days 0, 6, 12, and 18 according to a modified protocol (27). The experiment was carried out one hour after the injection was given. The Eddy Hot Plate Analgesiometer was utilized to confine the animals within a Perspex cylinder for the test. The plate temperature was maintained at 50.0 degrees Celsius, with a variation of 0.5 degrees. The response, whether it was paw stroking or jumping, along with the duration of pain, was recorded within a twenty-second timeframe to minimize the risk of tissue damage.

*Randall-selitto pressure test.*

Researchers adjusted the Randall-Selitto pressure test to assess static hyperalgesia (28). The Paw Pressure Analgesia Meter-PPA 01 measures the Paw Withdrawal Threshold (PWT). A minimal impact (<20 g) may trigger vocalization or paw disengagement as a pain response. On days 0, 6, 12, and 18, the test was performed one hour after administering the treatment.

*Von frey test*

The Von Frey test (IITC Electronic) was used to evaluate the sensitivity of mice to mechanical stimuli, including pressure or touch. The animals became acquainted with the experimental procedures through numerous sessions. The mice were placed in the testing chamber and given ten minutes to acclimate before the test began. Next, a Von Frey filament was utilized to exert pressure on the underside of the right hind paw. The paw withdrawal threshold (PWT) was observed, and the lowest pressure needed to trigger this response was measured and identified as the PWT. Three measurements were taken at three-minute intervals, with the experimenter unaware of the treatment being administered (29).

### Histopathological evaluation

After the *in vivo* experiment, the sciatic nerves from the animals were collected and preserved in neutral buffered formalin. Subsequently, the tissues were processed for Hematoxylin and Eosin (H&E) staining (Sigma Alrich), a standard histological method. A veterinary pathologist, unaware of the experimental treatments, examined the dyed slides subjected to H&E staining under a microscope. The severity of the histopathological lesions was evaluated on a scale from one to five, with one representing minor damage and five representing significant damage. The localization of these abnormalities-whether localized, multiple, or widespread-was documented by observing at both low (10x) and high (40x) magnification levels with a SOPTOP ICX41 microscope. The acquired photos were subsequently examined for analysis and measurements with Image-J software.

### In vivo anti-oxidant evaluation

The concentrations of reduced glutathione (GSH) and oxidized glutathione (GSSG) in sciatic nerve tissues were measured using a previously established method (30). The tissues were crushed in a phosphate buffer solution containing metaphosphoric acid and centrifuged at high speed to separate the clarified mixture. GSH levels were assessed by combining the homogenate with o-phthaldialdehyde (OPT) and measuring the resulting fluorescence. GSSG levels were assessed by treating the homogenate with N-ethylmaleimide (NEM) to inhibit free thiol groups, followed by incubation with OPT. Lipid peroxidation was assessed by measuring malondialdehyde (MDA) levels. MDA reacts with thiobarbituric acid (TBA) to form a measurable complex that can be detected using spectrophotometry. The concentration of MDA was determined using the Lambert-Beer equation (31).

### Statistical analysis

Analyses were conducted using GraphPad Prism 9. One-way ANOVA and Dunnett’s multiple comparison test were employed to assess significance. The values are presented as the means±SDs. A *P*-value less than 0.01 or 0.001 indicates significance.

## Results

### Physicochemical characterization of the TRE@NHA composites

**Figure 1:**
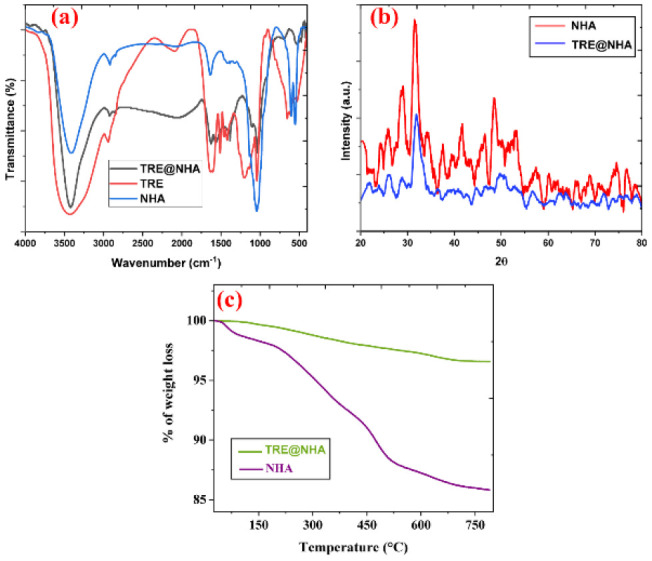
(a) FTIR spectrum; (b) XRD pattern; (c) TGA analysis of the prepared NHA and TRE@NHA-2

The integration of TRE into the NHA matrix was examined via a mixture of characterization methods, including FT-IR spectroscopy and HRTEM analysis. FTIR spectroscopy was used to identify the functional groups in the TRE@NHA composite (Figure 1a). The spectra displayed distinct peaks of each component. Peaks detected at around 3400 cm^-1^, 2800-3000 cm^-1^, 1623 cm^-1^, and 1594 cm^-1^ were ascribed to hydroxyl (OH) stretching, C-H stretching from aliphatic groups, and aromatic C=C stretching vibrations, respectively, so affirming the existence of alkaloid (berberine) in the turmeric root extract. Additionally, peaks at 537 cm^-1^, 619 cm^-1^, 902 cm^-1^, and 1030-1100 cm^-1^ were detected, indicative of the phosphate (PO43-) group in NHA. A wide band at around 3400 cm^-1^ and a sharp band at 630 cm^-1^ further confirmed the existence of hydroxyl (OH-) groups in the NHA structure. The FTIR study effectively validated incorporating TRE and NHA inside the composite material. The FTIR spectrum exhibited distinct peaks associated with both components, alkaloid (aromatic rings, carbonyl groups, and hydroxyl groups) and NHA (phosphate and hydroxyl groups), so offering compelling evidence for the composite’s production (32-33).

The XRD pattern of the synthesized NHA displayed distinct sharp peaks with 2θ values of around 25.79°, 31.94°, 34.35°, and 48.45°, indicative of its crystalline structure (Figure 1b). The peaks correspond to the (002), (211), (112), and (300) planes of the hydroxyapatite lattice, respectively. The XRD analysis of TRE@NHA confirmed the effective synthesis of NHA by revealing the presence of distinctive peaks. Nonetheless, the XRD pattern of TRE@NHA exhibited a reduction in peak intensity and sharpness relative to pure NHA. This data indicates a reduction in crystallinity, likely resulting from the interaction between the bioactive chemicals in the extract and the NHA surface (34). These interactions may have disrupted the crystal development process during the synthesis of TRE@NHA. The decreased crystallinity seen in TRE@NHA may be beneficial for certain biological applications. Reduced crystallinity often results in higher biocompatibility and bioactivity owing to greater surface area and improved ion exchange characteristics. This trait may facilitate the effective incorporation of the biocomposite into the biological environment and its ensuing interaction with adjacent tissues.

Thermogravimetric analysis (TGA) was used to ascertain the TRE concentration in the composite (Figure 1b). Both NHA and TRE@NHA demonstrated weight loss between 100 and 800 ^°^C, resulting from the elimination of volatile compounds. TRE@NHA exhibited an extra weight loss of around 0.5 wt% relative to pure NHA in the 400-800 ^°^C temperature range. The weight reduction is ascribed to the thermal deterioration of the included TRE, signifying its effective integration into the NHA matrix at a loading of around 0.5 wt%. This discovery is significant as it offers quantitative proof of the effective incorporation of TRE inside the NHA composite. Incorporating TRE into the composite is anticipated to provide superior biological attributes, including greater biocompatibility and antibacterial efficacy, which will be explored in future research.

**Figure 2:**
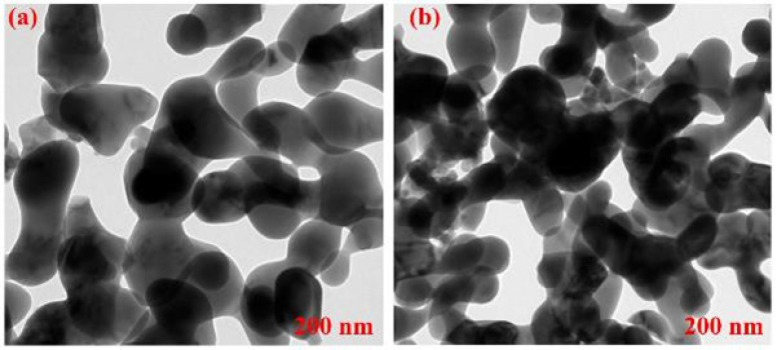
HRTEM images of the (a) nanohydroxyapatite (NHA) and (b) TRE@NHA prepared samples

High-resolution transmission electron microscopy (HRTEM) examination demonstrated a spherical shape for both NHA and TRE@NHA samples. A significant increase in grain size was seen in TRE@NHA compared to virgin NHA, as shown by detailed micrographs (Figure 2). This morphological change indicates a substantial impact of TRE on the NHA development process. The HRTEM study further validated the effective synthesis of the TRE@NHA composite, clearly illustrating the close interaction between TRE and NHA at the nanoscale. These data together indicate that TRE significantly affects the morphology and properties of NHA, as shown by the observed morphological and compositional alterations.

### In vitro cytotoxicity assessment

**Figure 3:**
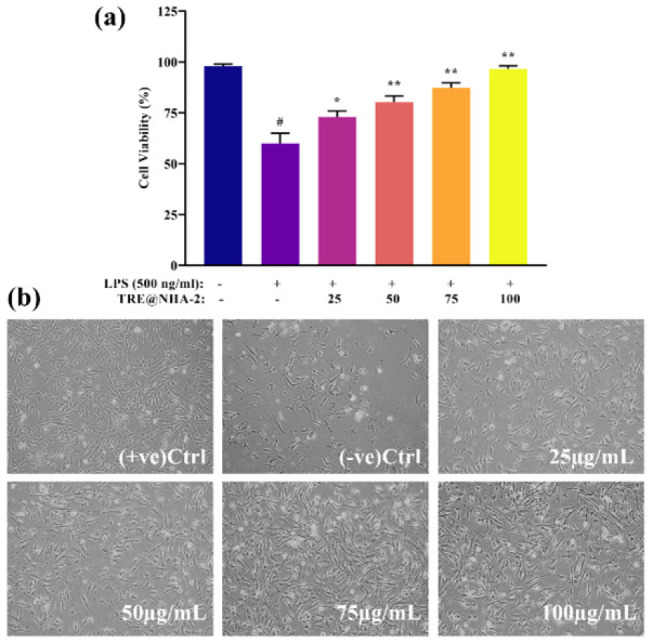
(a) Bar diagram representation of the cell viability of different concentrations TRE@NHA-1 on PC12 cells and (b) the cell morphology after 24 hr treatment on the cell culture

**Figure 4:**
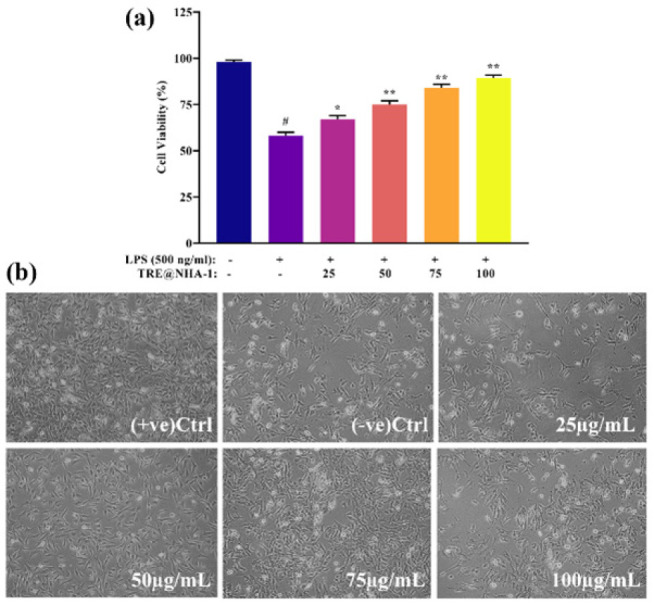
(a) Bar diagram representation of the effects of different concentrations of TRE@NHA-2 on the viability of PC12 cells and (b) the morphology of PC12 cells after 24 hr of culture

Cell viability experiments on LPS-stimulated PC12 cells were used to determine the biocompatibility of TRE@NHA-1 and TRE@NHA-2. Treatment with doses ranging from 25 to 100 μg/ml of either formulation did not significantly reduce cell viability compared to the control group, as assessed by the MTT test (Figures 3a-b and 4a-b). Microscopic examinations supported these results, revealing no obvious evidence of cell death or major morphological changes in cells treated with TRE@NHA-1 or TRE@NHA-2. Cell viability studies show that TRE@NHA-1 and TRE@NHA-2 are highly biocompatible at tested concentrations (25-100 μg/ml) in LPS-stimulated PC12 cells. The absence of considerable cytotoxicity, as shown by both MTT test results and microscopic observations, indicates that these formulations are well-tolerated by cells and do not cause major cellular damage.

### In vitro dual-staining assay

**Figure 5:**
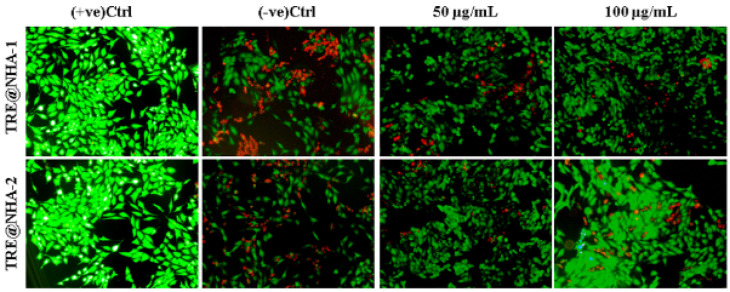
Fluorescence images of PC12 cells with AO/PI staining at 24 hr after the control, TRE@NHA-1 (50 µg/ml and 100 µg/ml) and TRE@NHA-2 (50 µg/ml and 100 µg/ml) treatments

A live/dead cell staining test using AO/PI was conducted on PC12 cells to assess the cytotoxic effects of TRE@NHA-1 and TRE@NHA-2. An examination of the stained cells under a microscope revealed that the group treated with TRE@NHA-2 exhibited a significantly greater number of healthy cells (green nuclei) compared to the group treated with TRE@NHA-1 (Figure 5). In contrast, the group treated with TRE@NHA-2 showed a significantly lower number of dead cells (red nuclei). The increased cell survival rate may be attributed to the higher concentration of beneficial compounds, including berberine, an anti-oxidant found in the tree turmeric root extract utilized to produce TRE@NHA-2 (35). Research has shown that berberine possesses protective properties, including the capacity to eliminate harmful reactive oxygen species and reduce cellular damage from oxidative stress (36).

The different levels of cell toxicity seen with TRE@NHA-1 and TRE@NHA-2 highlight the importance of adjusting formulation factors to enhance treatment effectiveness and reduce adverse effects. Based on the current findings, TRE@NHA-2 clearly shows greater potential as a therapeutic agent due to its enhanced safety features.

### Nuclear morphology assessment

**Figure 6:**
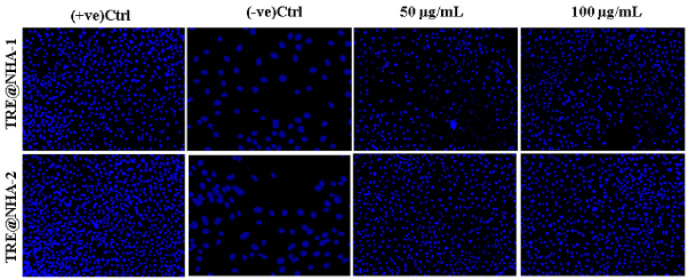
Fluorescence images of PC12 cells with 4′,6-diamidino-2-phenylindole (DAPI) staining at 24 hr after the control, TRE@NHA-1 (50 µg/ml and 100 µg/ml) and TRE@NHA-2 (50 µg/ml and 100 µg/ml) treatments

DAPI labeling was utilized to visualize the cell nuclei in PC12 cells and to detect indications of cell death (apoptosis). The control cells exhibited a typical nuclear structure with consistent DAPI staining, as shown in Figure 6. Exposure to LPS resulted in noticeable nuclear shrinkage, a typical cell death feature (apoptosis). The addition of TRE@NHA-1 or TRE@NHA-2 resulted in a reduction in the number of cells that died, which was directly proportional to the concentration of the treatment. A reduction in nuclear condensation confirmed this. Preventing LPS-induced nuclear condensation by TRE@NHA-1 and TRE@NHA-2 strongly supports their anti-apoptotic properties. The results are consistent with these substances’ previously documented cytoprotective properties (37). The superior apoptotic inhibition shown by TRE@NHA-2, in comparison to TRE@NHA-1, emphasizes its potential as a highly effective therapeutic intervention.

### In vitro mitochondrial membrane potential

**Figure 7:**
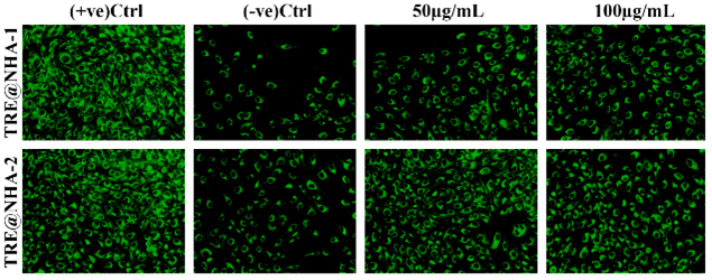
Fluorescence images of PC12 cells with Rhodamine 123 staining at 24 hr after the control, TRE@NHA-1 (50 µg/ml and 100 µg/ml) and TRE@NHA-2 (50 µg/ml and 100 µg/ml) treatments

The mitochondrial membrane potential (MMP) in PC12 cells treated with LPS was evaluated using rhodamine 123 labeling. The control cells exhibited a normal MMP with strong fluorescence inside the cell (Figure 7). Exposure to LPS resulted in a notable reduction in the MMP, as shown by a decrease in the Rhodamine 123 fluorescence intensity. The administration of TRE@NHA-1 or TRE@NHA-2 led to the restoration of MMP levels, which varied based on the dose. The treated cells demonstrated either partial or complete recovery, as illustrated in Figure 6. TRE@NHA-2’s superior ability to restore the MMP, compared to TRE@NHA-1, may have contributed to its enhanced cell survival. Mitochondrial activity is crucial for ATP generation, maintaining calcium balance, and regulating apoptosis (38). The protective effects of TRE@NHA-2 on mitochondrial integrity highlight its potential as a therapeutic agent for neurological diseases marked by mitochondrial dysfunction.

### In vitro anti-inflammatory assay

**Figure 8:**
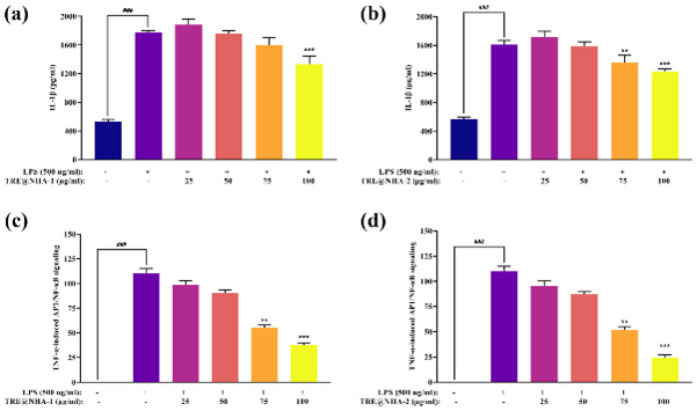
Quantitative representation of the levels of (a-b) IL-1β and (c-d) TNF-α differentiated PC12 cells in response to lipopolysaccharide (LPS)-induced inflammation

Cytokine inhibition was noted following the treatment of LPS-stimulated PC12 cells with TRE@NHA-1 or TRE@NHA-2 at doses between 25 and 100 μg/ml (Figure 8). The release of IL-1β, a proinflammatory cytokine, was significantly reduced (Figure 8 a-b). TRE@NHA-2 reduced TNF-α-induced NF-κB/AP-1 transcriptional activity in TNF-α cells in a dose-dependent manner. Significant inhibition was observed at doses between 25 and 100 μg/ml (Figure 8 c-d). The current study emphasizes the strong anti-inflammatory properties of TRE@NHA-2 in a regulated laboratory environment. TRE@NHA-2 has the potential to be utilized as a therapeutic treatment for inflammatory disorders, including CIPN, due to its ability to reduce the production of proinflammatory cytokines, such as IL-1β, and inhibit NF-κB activation.

The observed effects of TRE@NHA-2 on the generation of cytokines and the inhibition of NF-κB align with other studies that have shown the anti-inflammatory characteristics of this chemical. These findings provide compelling justification for further research on the therapeutic capabilities of TRE@NHA-2 in preclinical models of CIPN. The relationship between oxidative stress and inflammation in CIPN is well recognized. The demonstrated anti-oxidant and anti-inflammatory characteristics of TRE@NHA-2 indicate a possible dual mode of action in reducing CIPN disease.

### Experimental model and behavioral assessment

**Figure 9:**
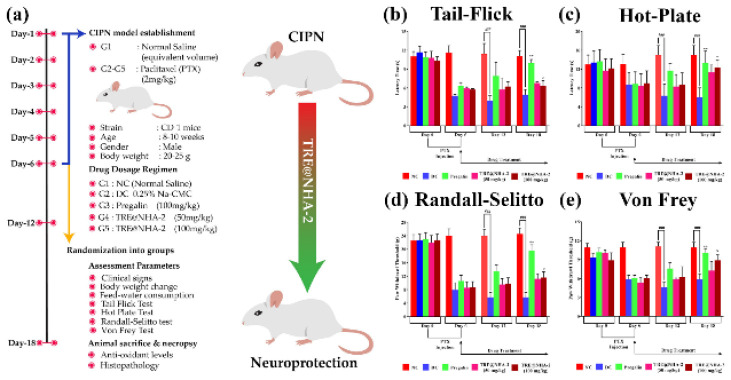
(a) Illustration of the animal experimental strategy. (b–e) Bar diagram representations of the effects of TRE@NHA-2 (50 µg/ml and 100 µg/ml) treatment on CIPN modulated nociceptive behaviors of the experimental animals, as determined through a tail flick (b), hot plate (c), Randall‒Selitto pressure (d), and Von Frey filaments (e)

TRE@NHA-2 was chosen as a potential candidate for future studies in an animal model of CIPN because of its promising *in vitro* findings showing considerable effectiveness against neuropathic pain. A mouse model of CIPN was created by delivering PTX to the rats. Nociception and allodynia were evaluated via behavioral methods such as the tail flick, hot plate, Randall-Selitto, and Von Frey tests (Figure 9).

Compared with the normal control (NC) group, the tail flip test revealed that PTX considerably decreased the time it took for the tail to flick, suggesting the presence of allodynia. Compared with the disease control (DC) group, both the pregabalin and the TRE@NHA-2 groups presented a prolonged delay in the pain control (DC) group starting from day 12, indicating an analgesic effect (Figure 9b). The hot plate test revealed that PTX caused heat hyperalgesia, as the reduced latency shows. The pregalin and TRE@NHA-2 groups presented longer delay periods than the DC group, suggesting decreased heat hyperalgesia (Figure 9c).

The Randall-Selitto test revealed that PTX caused a substantial reduction in the paw withdrawal threshold (PWT) compared with that of the control group (NC), demonstrating that allodynia is linked with neuroinflammation. Compared with the DC group, both the pregalin and TRE@NHA-2 groups presented elevated PWTs, indicating a decrease in mechanical allodynia (Figure 9d). Compared with the NC, the Von Frey test revealed that PTX significantly lowered the PWT, indicating mechanical allodynia. Pregalin and TRE@NHA-2 had greater PWT values than DC, indicating less mechanical allodynia (Figure 8e).

TRE@NHA-2 showed substantial effectiveness in reducing both heat hypersensitivity and mechanical pain caused by PTX. The efficacy of TRE@NHA-2 was equivalent to, or in some instances, surpassing that of conventional Pregalin, an anti-CIPN medication. Notably, TRE@NHA-2 did not cause any noticeable alterations in body weight or eating patterns. The present research offers convincing evidence of the effectiveness of TRE@NHA-2 in alleviating neuropathic pain caused by PTX. The observed decreases in heat sensitivity and mechanical stimuli sensitivity indicate that TRE@NHA-2 has a wide-ranging pain-relieving effect.

### Histopathological evaluation of sciatic nerve

**Figure 10:**
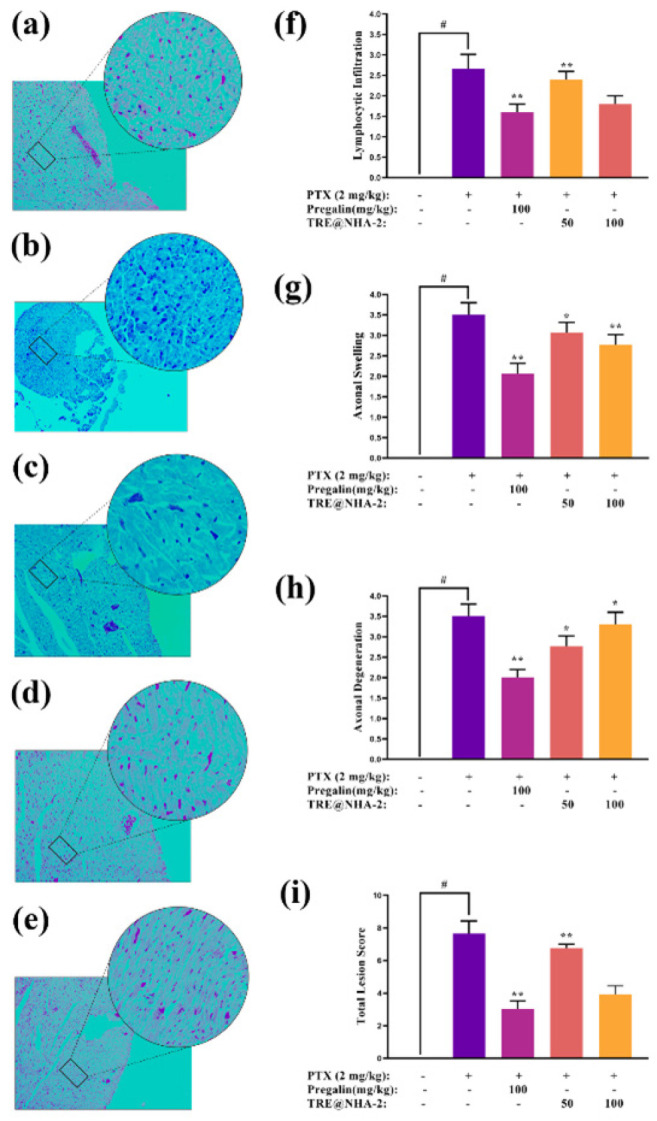
TRE@NHA-2 restores the CIPN-associated histopathological profile

Sciatic nerve slices were subjected to histological investigation to examine the neuroprotective effects of TRE@NHA-2 (Figure 10). In the control group, the sciatic nerves displayed preserved axons, Schwann cells, and myelin sheaths, and no pathological alterations were detected. The disease control (DC) group presented noticeable axonal degeneration, edema, and infiltration of lymphocytes in the sciatic nerves. The administration of Pregalin and TRE@NHA-2 resulted in a decrease in axonal degeneration and edema compared with those in the DC group. Although Pregalin substantially reduced these parameters, TRE@NHA-2 exhibited a similar pattern without achieving statistical significance. Compared with DC, pregalin significantly reduced lymphocytic infiltration. At a 100 mg/kg dose, TRE@NHA-2 also dramatically decreased infiltration, reaching levels similar to those in the pregalin group. The lower dosage of TRE@NHA-2 tended to decrease; however, this decrease did not reach statistical significance. The total lesion score, which reflects overall histopathological damage, was considerably lower in the Pregalin and TRE@NHA-2 (100 mg/kg) groups than in the DC group.

The histopathology data strongly corroborate the neuroprotective benefits of TRE@NHA-2 in reducing PTX-induced neuropathic damage. The decreases in axonal degeneration, edema, and lymphocytic infiltration indicate that TRE@NHA-2 has positive benefits via many routes, such as anti-inflammatory and neurodegenerative properties.

### Oxidative stress markers in sciatic nerve tissue

**Figure 11:**
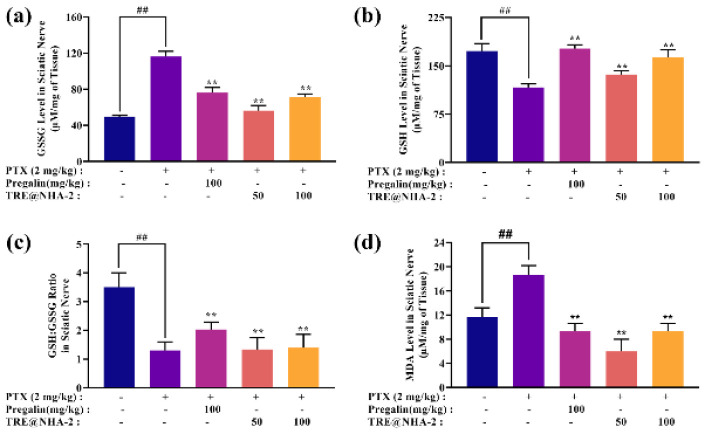
TRE@NHA-2 has *in vivo* antioxidative properties

To evaluate the oxidative stress linked to CIPN and the possible anti-oxidant properties of TRE@NHA-2, the concentrations of oxidized glutathione (GSSG) and glutathione (GSH) and the ratios of GSH to GSSG and malondialdehyde (MDA), were quantified in sciatic nerve tissue (Figure 11). PTX administration led to a significant increase in GSSG levels, confirming the presence of oxidative stress. While Pregalin did not significantly decrease, both doses of TRE@NHA-2 led to a significant decrease in GSSG levels compared with those in the DC group. This study revealed that PTX-induced CIPN led to considerable decreases in GSH levels, providing further evidence of oxidative stress. Both the Pregalin and TRE@NHA-2 treatments led to increased GSH levels. The increase was statistically significant only in the Pregalin group.

The ratio of reduced GSH to GSSG was significantly lower in the DC group than in the NC group, indicating the presence of oxidative stress. The administration of Pregalin and TRE@NHA-2 resulted in a considerable increase in this ratio, indicating an increase in the redox state. The administration of PTX resulted in a considerable increase in MDA levels compared with those in the NC group, suggesting an increase in lipid peroxidation. Compared with DC alone, both pregalin and TRE@NHA-2 effectively decreased the MDA level. The increase in MDA and GSSG levels, the decrease in GSH levels, and the GSH:GSSG ratio are evidence of oxidative stress in the sciatic nerve tissue of rats with PTX-induced CIPN. These results align with other studies that have shown the involvement of oxidative stress in the development of CIPN (39-40). TRE@NHA-2 has a significant ability to reduce MDA and GSSG levels significantly while also increasing GSH levels. The GSSG ratio reflects the anti-oxidant properties of the substance. These findings suggest that the neuroprotective effects of TRE@NHA-2 may be attributed in part to its ability to alleviate oxidative stress in the peripheral nervous system. The anti-oxidant effects of TRE@NHA-2 are similar to those of Pregalin, indicating its potential as a promising therapeutic option for CIPN management.

## Discussion

The present research highlights the effective incorporation of TRE into the NHA matrix, leading to the creation of TRE@NHA composites. The physicochemical analysis of the composites indicated that the integration of TRE did not substantially modify the crystalline structure of NHA but resulted in morphological alterations, indicating a possible impact on the material’s qualities.

*In vitro* investigations underscored the safety and therapeutic efficacy of TRE@NHA composites, especially TRE@NHA-2. The composite demonstrated notable anti-inflammatory and neuroprotective properties, as shown by its capacity to suppress cytokine production, diminish oxidative stress, and enhance neuronal survival. The enhanced effectiveness of TRE@NHA-2 over TRE@NHA-1 is due to the bioactive chemicals in the tree turmeric root extract, including berberine, known for its anti-oxidant and anti-inflammatory effects.

The *in vivo* investigation further validated the therapeutic efficacy of TRE@NHA-2 in a rat model of chemotherapy-induced peripheral neuropathy (CIPN). The combination significantly reduced neuropathic pain, as shown by improvements in behavioral assessments. Histopathological examination demonstrated less neuronal injury and reduced inflammation in the sciatic nerve of rats treated with TRE@NHA-2. Furthermore, the composite had substantial anti-oxidant properties, as shown by the decrease in oxidative stress indicators.

The results indicate that TRE@NHA-2 may serve as a potential therapeutic option for managing CIPN. The composite’s dual role as an anti-inflammatory and anti-oxidant agent may enhance its effectiveness in alleviating neuropathic pain and facilitating neural repair. Additional research is necessary to clarify the underlying mechanisms of action and to investigate the long-term significance of TRE@NHA-2 therapy.

The cells were photographed via a fluorescence microscope at 10X magnification

The cells were photographed via a fluorescence microscope at 10X magnification

The cells were photographed via a fluorescence microscope at 10X magnification

Data is represented as mean±SD from triplicate data of three independent experiments. Statistical significance was analyzed through one-way ANOVA followed by Dunnett’s multiple comparison tests and represented as ## for *P<*0.001, significantly different from the normal cells, untreated with LPS, TRE@NHA-1, TRE@NHA-2, or as ** and *** for *P<*0.01 and *P<*0.001, respectively, when the comparison was with the TRE@NHA-1, TRE@NHA-2 untreated by LPS treated group

The data are presented as the means±SDs, and statistical significance is denoted as ** and *** or ### for* P-values<*0.05, 0.01, and 0.001, respectively. P-values are shown with either ‘*’ or ‘#’ depending on whether the comparison was with the NC, DC, or Pregalin group

[a–e] Representative images showing H&E-stained sciatic nerve sections from the NC (a), DC (b), pregalin (c), 50 µg/ml (d), and 100 µg/ml (e) groups at low (10X; scale bar: 100 μm) and high (40X; scale bar: 20 μm) magnifications. (f–i) Quantitative representation of scorings for axonal degeneration (f), axonal swelling (g), lymphocytic infiltration (h), and overall lesions (i)

The data are presented as the means±SDs; n=6 in each experimental group. The statistical significance of the observations was determined through one-way ANOVA followed by Dunnett’s multiple comparison test and is represented as ## for *P<*0.001, which is significantly different from that of the NC group; * for *P<*0.01 and ** for *P<*0.001, which is significantly different from that of the DC group; and #, which is comparable to that of the Pregalin group

[a-d] Quantitative representations of the antioxidant effects of TRE@NHA-2 on neuropathic sciatic nerves, which include the levels of GSSG (a), GSH (b), the GSH:GSSG ratio (c), and MDA (d)

The data are presented as the means±SDs; n=6 in each experimental group. The statistical significance of the observations was determined through one-way ANOVA followed by Dunnett’s multiple comparison test and is represented as ## for *P<*0.001, which is significantly different from that of the NC group; * for *P<*0.01 and ** for *P<*0.001, which is significantly different from that of the DC group; and #, which is comparable to that of the Pregalin group

## Conclusion

This study provides excellent evidence for the therapeutic effectiveness of TRE@NHA-2 in treating chemotherapy-induced peripheral neuropathy (CIPN). The *in vitro* findings showed that TRE@NHA-2 exhibited anti-inflammatory and neuroprotective properties, as shown by its ability to inhibit proinflammatory cytokine generation, reduce oxidative stress, and increase cell survival. In an *in vivo* model of chemotherapy-induced peripheral neuropathy, TRE@NHA-2 markedly reduced heat and mechanical hypersensitivity, comparable to the standard treatment drug pregabalin. A histopathological examination revealed significant reductions in nerve damage, including axonal degeneration, edema, and inflammatory cell infiltration. Furthermore, TRE@NHA-2 had considerable anti-oxidant activity, as seen by lower oxidative stress markers in the sciatic nerve. The findings suggest that TRE@NHA-2 might be a feasible therapeutic option for CIPN, possibly improving patient quality of life and reducing the burden of this painful disease.

## Data Availability

Data will be made available upon request.

## Declaration

We have not used any AI tools or technologies to prepare this manuscript.
